# Expression, purification and kinetic characterization of recombinant benzoate dioxygenase from *Rhodococcus ruber *UKMP-5M

**Published:** 2016-09

**Authors:** Arezoo Tavakoli, Ainon Hamzah, Amir Rabu

**Affiliations:** 1 Islamic Azad University Eghlid branch, Fars, Iran; 2 School of Biosciences and Biotechnology, Faculty Science and Technology, University Kebangsaan Malaysia, Selangor, Malaysia

**Keywords:** Benzoate dioxygenase, *Rhodococcus* ruber, Purification, GC-MS analysis

## Abstract

In this study, benzoate dioxygenase from *Rhodococcus ruber *UKMP-5M was catalyzed by oxidating the benzene ring to catechol and other derivatives. The benzoate dioxygenase (*ben*A gene) from *Rhodococcus ruber *UKMP-5M was then expressed, purified, characterized, The *ben*A gene was amplified (642 bp), and the product was cloned into a pGEM-T vector. The recombinant plasmid pGEMT-benA was digested by double restriction enzymes *BamH*I and *Hind*III to construct plasmid pET28b-benA and was then ligated into *Escherichia coli *BL21 (DE3). The recombinant *E. coli *was induced with 0.5 mM isopropyl *β*-*D*-thiogalactoside (IPTG) at 22˚C to produce benzoate dioxygenase. The enzyme was then purified by ion exchange chromatography after 8 purification folds. The resulting product was 25 kDa, determined by sodium dodecyl sulphate polyacrylamide gel electrophoresis (SDS-PAGE) and western blotting. Benzoate dioxygenase activity was found to be 6.54 U/mL and the optimal pH and temperature were 8.5 and 25°C, respectively. Maximum velocity (Vmax) and Michaelis constant (Km) were 7.36 U/mL and 5.58 µM, respectively. The end metabolite from the benzoate dioxygenase reaction was cyclohexane dione, which was determined by gas chromatography mass spectrometry (GC-MS).

## INTRODUCTION

Aromatic hydrocarbons such as BTEX (benzene, toluene, ethylbenzene, and xylenes) which are found in crude oil, are used in many industrial products and known to be pollutants. They can seep through the soil and groundwater from underground storage tanks and pipeline leakages or waste disposal practices and contaminate water and groundwater [1]. The Environmental Protection Agency (EPA) classified BTEX as priority pollutants and revealed their toxic effects on living organisms and environments [2]. Reducing and cleaning up hydrocarbons from the environment is thus considered to be a very important process, and the remediation of contaminated sites has received special attention. Compared with physical or chemical methods, microbial degradation is one of the most effective and economical solutions for the removal of aromatic hydrocarbons from contaminated environments [3]. Microbial enzymes such as hydrocarbon dioxygenase can be valuable for such biodegradation processes. Aromatic hydrocarbon dioxygenases have been shown to have an important role in the biodegradation of BTEX by the intradiol or extradiol cleavage of the aromatic ring. These enzymes are identified in many bacteria such as *Rhodococcus*, *Pseudomonas*, and can degrade hydrocarbons to less toxic compounds such as catechol [4,6].

Since the benzene ring cleavage is essential for the aromatic hydrocarbon degradation process, benzoate and its derivatives are common compounds that can be produced through aerobic and anaerobic hydrocarbon biodegradation [3]. Under aerobic conditions, the ring is cleaved by adding oxygen through an oxidation reaction; hence, different intermediate compounds such as arene *cis*-diols could be obtained [7, 8]. The production of *cis-diols *by enzymes such as toluene dioxygenase, naphthalene dioxygenase and biphenyl dioxygenase which are known as Rieske type non-heme iron oxygenases*, *has been previously reported [9]. During the next step, catechol can be produced and the reaction that follows can lead to Kerbs cycle intermediates. The aerobic degradation of benzoate has been elucidated in various bacteria [3]. Benzoate dioxygenase from *Acinetobacter calcoaceticus *ADP1 and *P. putida *have been shown to catalyze the dihydroxylation of benzoates [5,10,11].

Many genes such as *xyl, bop *and *ben *are involved in the dihydroxylation benzoate ring, encode various dioxygenases by plasmids or chromosomes, and yield a nonaromatic *cis*-diol to be converted into catechol [6]. The *ben*A gene is involved in benzoate metabolism in many *Rhodococcus *strains [12]. Because of their high catabolic diversity for hydrocarbon degradation, *Rhodococcus *species are frequently isolated from contaminated environments and widely used in bioremediation [3]. In Malaysia, *Rhodococcus ruber *UKMP-5M was isolated from oil contaminated soil, whereas other studies showed the bacterium to be capable of degrading crude oil and toluene [13]. The activity of catechol dioxygenase shows that *R. ruber *UKMP-5M can degrade catechol by catechol 2,3 dioxygenase using a *meta *pathway to produce 2-hydroxymuconic acid [14]. The present study focused on the expression, purification and characterization of benzoate dioxygenase from *Rhodococcus ruber *UKMP-5M. The results can help characterize a functional gene for the bioremediation of polluted environments.

## MATERIALS AND METHODS

**DNA amplification and cloning: **Total DNA from *Rhodococcus ruber *UKMP-5M was extracted by a Wizard genomic DNA purification kit (Promega) and analyzed using 1% agarose gel and a spectrophotometer. A specific primer was designed based on genome sequences from *R. ruber *UKMP-5M with two restriction sites *BamH*I and *Hind*III to amplify the *ben*A gene in an automated thermal cycler (Bio-Rad). We used the Forward 5-'GGA TCC ATG GAA CTG GAC CGA GGT CCA GCA GGT GC-3' and Reverse:5'-AAGCTTTCAGAAGGGGTAGACGGGCACGTCGCG-3' primers.

The PCR product (~ 0.6) was purified using a QIA Miniprep purification kit (Qiagen). The purified benA fragment was ligated into a pGEM®-T Easy vector by T4 DNA ligase (Promega) and transformed into competent cells of *E. coli *DH5α after being heat shocked at 42ºC for 50s. The cells were grown on LB agar containing ampicillin (Sigma) (50µg/ml), 50 mg/mL 5-bromo-4-chloro-3-indoyl-*β*-*D-*Galactopyranoside (X-Gal) (Promega) and 100 mM isopropyl *β*-*D-*1-thiogalacto pyranoside (IPTG) (Sigma), and the positively transformed *E. coli *was screened by PCR. The recombinant plasmid pGEMT- benA was extracted via QIAprep Miniprep kit (Promega) and analyzed using 1% agarose gel. The pGEMT-benA and pET 28b vector (Novagen) were digested using *BamH*I and *Hind*III (Promega) and the purified fragment benA was inserted into pET 28b by T4 DNA ligase after incubation at different temperatures (14-16°C) for 14-17 hours. The product was then transformed into *E. coli *DH5α using heat shock, the cells were cultured on LB agar supplemented with kanamycin (50 µg/mL), and the recombinant plasmid pET 28b- benA was extracted. The DNA sequences of pGEMT-benA and pET 28b-benA were evaluated using specific benA and universal primers and the data were analyzed using BLAST and VecScreen bioinformatics software.

**Growth of recombinant** *E. coli* **: **The pET 28b-benA was transformed into *E. coli *BL21 (DE3). A pre-culture from the transformed *E. coli *was prepared and incubated at 37ºC to reach optical density ~ 0.5 at OD550nm. The standard inoculum (10%) was added to Minimal salt medium (MSM) [13] supplemented with (0.5-2.5 mM) sodium benzoate. The culture was incubated at 30°C, 150 rpm for 72 hours and the growth was measured at OD550nm. The control was prepared with the same condition as *E. coli *but without the plasmid.

**Expression and purification: **A single colony of *E. coli *BL21 (DE3) containing plasmid pET 28b-benA was inoculated into 10 mL LB broth containing 50 µg/mL kanamycin and incubated at 37ºC, 250 rpm for 16 hours. The culture was centrifuged at 4ºC, 4000 rpm for 15 minutes and the pellet was diluted 5-fold (50 mL) with pre-warmed LB broth. The cells were incubated with shaking to obtain an OD550nm of 0.5-0.7. Afterward, 1 mL of the culture was collected as a non-induced sample and the medium was cooled to 22ºC to limit the formation of inclusion bodies. The expression of benzoate dioxygenase was induced by adding 0.5 mM IPTG and taking it out after 2, 4, 6 and 16 hours. The cells were harvested by centrifugation at 13000 rpm, 4°C for 20 minutes. The pellet was washed and resuspended in a lysis buffer (50 mM NaH2PO4 with 300 mM NaCl) (pH 8.0) containing 1 μg/mL lysozyme, and 0.1 mM/mL phenylmethyl sulfonyl fluoride (PMSF) (Sigma) was added to protect the protein structure. The mixture was then incubated in ice for 30 min. Cell disruption was performed by a sonicator (Sonics-vibra cell) at 20s pulses with 5 minutes’ rest for 6 times. The suspension was centrifuged at 4°C, 12000 rpm for 40 minutes. The resulting supernatant and pellet were analyzed by SDS-PAGE. The expression of His-tag protein was confirmed by western blot analysis using a monoclonal antibody (Antibody Histidine-Alkaline Phosphatase) (Sigma). The over expression of benzoate dioxygenase was carried out in a large scale (at least 2L) and the supernatant was filtered using a 0.22 µm membrane filter. The protein was purified by ion exchange chromatography (IXC) using an AKTA prime plus system (No 1314455 Sweden) (GE Health care) according to the manufacturors’ instructions. The machine and Hi Trap SP-FF Sepharose column (i.d×h 0.7×2.5 cm) were washed stepwise with buffer A (wash buffer) which contained 50 mM acetic acid (pH 4.3) or a 50 mM HEPES buffer (pH 8.0) at a flow rate of 0.8 mL/min. The crude protein was eluted in buffer B (elution buffer) containing 200 mL washing buffer with 0-1.0 M NaCl supplemented with 0.5-1 mM dithiothreitol (DTT) or mercaptoethanol (ME). In the last step, the machine and column were re-equilibrated with buffer A. The collected fractions from bound and unbound proteins were analyzed using SDS-PAGE and western blotting to determine the molecular weight and purity of the target protein. The concentration of benzoate dioxygenase was determined using a Bicinchoninic acid (BCA) kit at OD562nm. After purification, the desired protein was concentrated by a vivaspin or Amicon (Millipore) protein column to the maximum volume of 3 mL and kept at -20ºC.

**Enzyme characterization: **The reaction of benzoate dioxygenase was measured by converting reduced *β*-Nicotinamide adenine dinucleotide (NADH) to *β*-Nicotinamide adenine dinucleotide (NAD) by a spectrophotometer at 340nm. All assays were carried out in triplicates. The reaction mixture was prepared as follows: 50 mM sodium/potassium phosphate (110 µL), 0.1 mM FeSO4 (11 µL), sodium benzoate as a substrate (1.1 µL), 2 mM reduced *β*-Nicotinamide dinucleotide phosphate (3.5 µL) and purified benzoate dioxygenase (100 µL).The reaction mixture was adjusted to 1.1 mL by adding H2O (784.4µL). The reaction was monitored at 1 minute intervals for 10 minutes at 25°C by sa pectrophotometer at 340 nm. The enzyme activity was calculated using a general equation based on unit/mL, where one unit is the amount of enzyme that catalyzes the conversion of 1.0 µmol of substrate to the expected product per minutes at a standard assay condition [15].

**Optimal pH and temperature: **Optimum pH was determined in standard assay conditions as described above, except that the following buffer systems from 6.0 to 11.0 were used: 50 mM sodium/potassium phosphate (pH 6-7.5), Tris (hydroxylmethyl) methylamine (pH 8-9.5) and NaHCO3 (pH 10-11) at 0.5 intervals. The mixture was incubated for 3 minutes at 25ºC and OD340nm was measured. At optimal pH, the temperature was adjusted to 4, 20, 25, 37, 40, 50, 60, 70 and 80°C for the reaction mixture. The enzyme activity was measured after 3 minutes of incubation by spectrophotometry at OD340nm.

**Determination of kinetic parameters: **The effect of substrate concentration on enzyme activity was determined by maximum velocity (Vmax) and Michaelis constant (Km) by varying the concentration of sodium benzoate at a range of 0.1-1.2 mM to the total adjusted volume of 1.1 mL using water. The mixture was incubated for 3 minutes at 25°C and NAD production was measured at OD340nm. The Km and Vmax were calculated using Lineweaver Burk plots.

**Substrate specificity: **Different substrates (benzene, benzoic acid, butyl benzene and ethyl benzene) at 1 mM concentration were replaced with sodium benzoate and the efficiency of each substrate for benzoate dioxygenase activity was measured at OD340nm.

**Analysis of metabolites: **The mixture was prepared in a total volume of 1 mL and the reaction was stopped after 5 minutes of incubation by adding 300 µL of 0.1 M HCl. Protein was removed from the mixture using vivaspin 500 (Sartorious, Germany) to add 300 µL diethyl ether. One microliter from the upper layer was injected into the injection port of the gas chromatography-mass spectrometry (GC-MS) system [16].

## RESULTS AND DISCUSSION

*Rhodococcus ruber *UKMP-5M has the ability to degrade many aromatic hydrocarbons including toluene and catechol [14]. In this research, benzoate dioxygenase from *R. ruber *UKMP-5M was purified and the kinetic characteristics were determined. Benzoate and its derivatives were degraded via hydroxylation by incorporating two oxygen atoms into the aromatic nucleus [17].

The *ben*A gene from *R. ruber *UKMP-5M was amplified at 64°C, yielding a product of 0.6 kb. The recombinant plasmid pGEMT-benA was 3.6 kb. Sequence analysis of the benA fragment showed 70% similarity with the aromatic ring dioxygenase using *Rhodococcus equi *103S. The pGEMT-benA plasmid was digested to two fragments; the linear pGEMT fragment (~3000 bp) and the inserted fragment benA (~600 bp). Plasmid pET 28b-benA was constructed successfully into *E. coli *DH5α after incubation for 14 hours at 15°C. The recombinant pET 28b-benA was ~ 6 kb which was included in pET 28b (5.4 kb) and the benA fragment (0.6 kb). The accuracy of the benA fragment was confirmed by PCR amplification and sequencing analysis.

The highest growth of *E. coli *BL21 containing pET 28b-benA was determined when cells were incubated in 0.5 mM sodium benzoate for 24 hours, as compared to 1 and 2.5 mM of sodium benzoate. In *Klebsiella oxytoca *C302 the highest growth at 0.5 mM benzoate occurred after 3 hours of incubation [18]. The highest growth in *Rhodococcus *sp LIN was observed after 33 hours of incubation in 2 mM benzoate [3]. For *S. marcescens *DS-8, the highest growth was observed after incubation in 5 mM of sodium benzoate for 72 hours [19].

**Figure 1:**
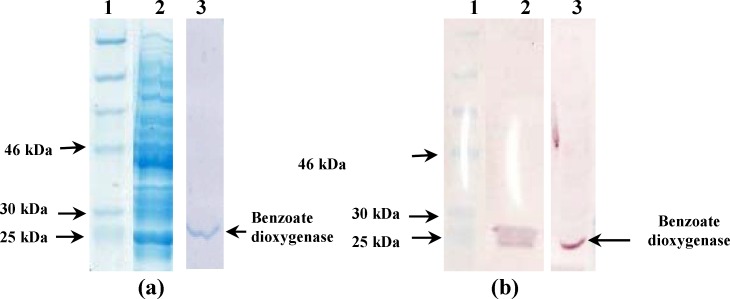
SDS-PAGE and western blot analysis of benzoate dioxygenase. (a) SDS-PAGE analysis before and after purification. Lane 1: Molecular mass marker, Lane 2: soluble fraction induced with IPTG at 6 hours, Lane 3 Purified benzoate dioxygenase. (b) Western blotting before and after purification. Lane 1: Molecular mass marker, Lane 2: Expression product of the plasmid pET28b-benA after induced for 6 hours, Lane 3: Purified benzoate dioxygenase

The transformed *E. coli *BL21 (DE3) was induced with 0.5 mM IPTG at 22°C, 250 rpm after 6 hours. The molecular weight of benzoate dioxygenase was 25 kDa (Figure 1a, b lane 2) and the isoelectric point (pI) was 10.38. The obtained protein was purified and eluted with an NaCl concentration of 1.0 M that showed into flow-through fractions 11- 12 of column. The purified benzoate dioxygenase was observed as a single band on the SDS-PAGE and western blot (Fig.1a,b lane3). The concentration of purified benzoate dioxygenase was 1.71 mg/mL, purified at 8 folds with a 62% yield by ion exchange chromatography (Table 1).

**Table 1:** Summary of benzoate dioxygenase purification from *R. ruber* UKMP-5M

**Purification stage**	**Volume (mL)**	**Total activity (Unit)**	**Total protein (mg)**	**Specific activity (U/mg)**	**Yields (%)**	**Purification folds**
**Cell-free extract**	30	539	218	2.47	100	1
**Ion exchange chromatography**	10	334	17.1	19.6	62	8

The highest benzoate dioxygenase activity was 6.54 U/mL, which was close to the benzoate dioxygenase activity by *P. putida *(6.67 U/mL). The benzoate dioxygenase activity by *P. arvilla *C-l was 4.2 U/mg [20]. The reported benzoate dioxygenase activities of *A. calcoaceticus *ADP (18.2 U/mg) [5], *P. putida *ML2 (20 U/mg) [21], and *P. putida *sp (58 U/mg) [15] were higher than that of *R. ruber *UKMP-5M. The benzoate dioxygenase activity of *Amycolatopsis *species such as *A. rugosa *strains DSM 43194, DSM 43387 and DSM 43388, was in the range of 12-53 U/mg [22], which is slightly higher than what was observed in the present study. Similar to other studies, the cofactor NADH and exogenous Fe+2 were necessary for the benzoate dioxygenase activity of *R. ruber *UKMP-5M [15, 23, 24]. A marked concentration-dependence for crude and purified benzoate dioxygenases demonstrated that benzoate dioxygenase activity increased when the reaction mixture was incubated with successive portions of substrate [15].

**Figure 2:**
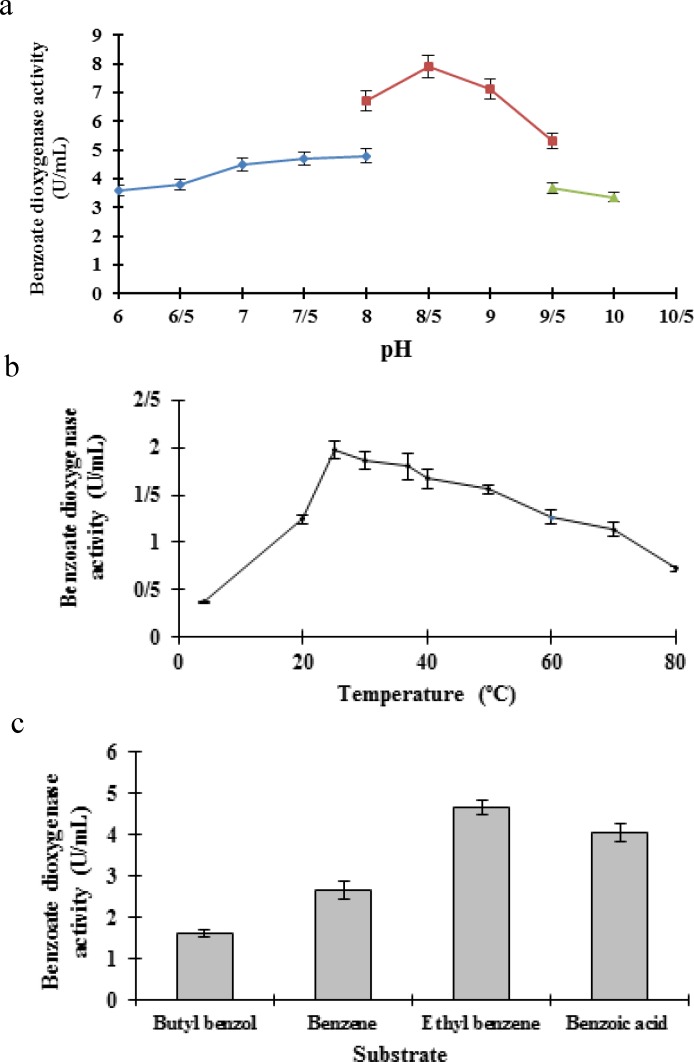
Effect of kinetic parameters for benzoate dioxygenase activity (a) Effect of different pH by using the following buffers:  ) 50 mM K2HPO4 (pH 6-8),  ) 50 mM Tris-buffer (pH 8-10),  ) 50 mM NaHCO3 (pH 10-11). The incubation time was three minutes. (b) Effect of different temperature (c) Effect of different substrates for benzoate dioxygenase activity

The optimal pH for benzoate dioxygenase activity was found to be 8.5 (Figure 2 a), which was close to the finding of other studies regarding *Arthrobacter *and *Rhodococcus *strains [25]. The optimal pH of *S. marcescens *DS-8 [19] and *Hydrogenophaga *(Betaproteobacteria) was 8.0 [20]; however, the optimum pH for *K. oxytoca *C302 [25] and *P. arvilla *C1 [18] were 7.0 and 6.7, respectively, which were both less than the optimal pH for the benzoate dioxygenase activity of *R. ruber *UKMP-5M.

For *R. ruber *UKMP-5M, the optimal temperature for benzoate dioxygenase activity was 25°C and the enzyme activity decreased considerably at any temperature lower than 20°C or higher than 60°C (Fig. 2b). The optimal temperatures for benzene dioxygenase activities of *K. oxytoca *C302 and *P. arvilla *C1 were 30°C and 24°C, respectively [18, 25]. The Vmax and Km values for benzoate dioxygenase of *R. ruber *UKMP-5M were 7.36 U/mL and 5.58 µM, respectively. The km for benzoate dioxygenase of *A. calcoaceticus *ADP1 (26 µM) [26] and *P. putida *(32 µM) [5] were much higher than the Km value of* ruber *UKMP-5M, but the Km value of *P*. *arvilla *C1 (3 µM) was less than *R. ruber* UKMP-5M [27].

Benzoate dioxygenase has a role in many hydrocarbon pathways because it can be used in various substrates [12]. The recombinant benzoate dioxygenase from *R. ruber *UKMP-5M was able to utilize benzene, ethyl benzene and benzoic acid as well as sodium benzoate (Fig.2c). Several studies have reported that *B. subtilis *strain MO5, *Rhodococcus rhodochrous *strain DSM 43274T, *Pseudomonas stuzeri *11C2 [21], *Rhodococcus *sp. strain DK17 [24] and *A. calcoaceticus *ADP1 [5] used similar substrates. However, higher enzyme activity was observed by the benzoate substrate [5].

*Identification of reaction products: *The end metabolite of the reaction was cyclohexamine dione that was observed in GC-MS. In the *Rhodococcus *strain, 19070 benzoate was converted to non-aromatic *cis*-diol [28]. The benzene dioxygenase of *P. putida *ML2 catalyzed the double hydroxylation of benzene to produce *cis*-1, 2- dihydroxycyclohexa-3, 5-diene [21]. The *ben*A gene was able to cleave with the benzene ring in substrates such as benzene and sodium benzoate; however, several genes were needed to produce catechol [29].

The amino acid sequence of benzoate dioxygenase from *R. ruber *UKMP-5M showed a 53% similarity with benzene 1, 2 dioxygenase of *P. putida *F1 and a 60% similarity with *Mycobacterium vanbaalenii *PYR-1. The homology for benzoate dioxygenase *R. ruber *UKMP-5M with biphenyl dioxygenase from *P. putida *F1 and terminal dioxygenase *R. erythropolis *were 47% and 60%, respectively. The benzoate dioxygenase family showed more sequence similarity with aromatic dehydrogenases such as biphenyl and toluene dioxygenase [30, 31]. Benzoate dioxygenase oxidizes aromatic hydrocarbons and related compounds by hydroxylationg the dioxygenase ring to catalyze dioxygenation reactions [32, 33]. *R. ruber *UKMP-5M benzoate dioxygenase functions in different pathways where the *ben*A gene assists other genes to convert benzoate to catechol. The results of our study indicate that this bacterium has the ability to biodegrade other BTEX, because the benzoate dioxygenase was able to use benzene and ethyl benzene as substrate. These abilities suggested that *R. ruber *UKMP-5M possesses an aromatic ring-hydroxylating dioxygenase similar to other aromatic hydrocarbon dioxygenases in *Pseudomonas *and *Acinetobacter *encoded by *xyl or ben *genes*.*
